# Innovative Impression Technique for Microstomia Induced by Epidermolysis Bullosa

**DOI:** 10.7759/cureus.103954

**Published:** 2026-02-20

**Authors:** Hasnaa Rokhssi, Faiza Benfdil, Leila Fajri

**Affiliations:** 1 Department of Prosthodontics, Faculty of Dental Medicine, Mohammed V University, Rabat, MAR

**Keywords:** epidermolysis bullosa, impression, limited mouth opening, microstomia, removable partial denture

## Abstract

A removable partial denture (RPD) is a prosthesis that requires a high degree of precision during impression making. Adequate mouth opening is essential during impression procedures to ensure proper placement and orientation of the impression tray.

However, this requirement poses a challenge in patients with microstomia, necessitating modification of conventional impression techniques to complete this crucial step in prosthesis fabrication.

This report describes a modified impression technique for fabricating a conventional RPD in a patient with epidermolysis bullosa and restricted mouth opening. A flexible tray reinforced with composite fibers was used to obtain the preliminary maxillary impression, followed by a definitive impression made using a customized two-part resin-silicone tray.

## Introduction

Microstomia is defined as a reduced oral aperture [1]. Limited mouth opening may result from burns, trauma, microinvasion, temporomandibular joint (TMJ) dysfunction, surgical management, treatment of orofacial neoplasms, head and neck radiotherapy, reconstructive lip surgeries, cleft lip, or systemic and inherited conditions such as epidermolysis bullosa [2,3].

Epidermolysis bullosa (EB) comprises a group of rare, genetically inherited disorders characterized by blistering of the skin and mucous membranes, frequently followed by scarring after minimal trauma [4,5].

This dermatological condition leads to significant tissue fragility, posing challenges during dental treatment and prosthetic rehabilitation [6].

Epidermolysis bullosa (EB) comprises four principal types that differ in both phenotype and genotype: epidermolysis bullosa simplex (EBS), junctional epidermolysis bullosa (JEB), dystrophic epidermolysis bullosa (DEB), and Kindler syndrome, with over 30 recognized clinical subtypes [7]. In epidermolysis bullosa, mucocutaneous fragility results from impaired structural integrity of proteins responsible for epidermal mechanical stability and dermoepidermal adhesion. These defects involve key components of the keratin cytoskeleton and the basement membrane zone, which are essential for maintaining epithelial cohesion and anchorage to the underlying connective tissue. Pathogenic variants affecting multiple structural proteins account for the marked genetic heterogeneity of the disorder [8].

Transmission electron microscopy (TEM) is considered the gold standard for establishing the diagnosis of EB [9].

Epidermolysis bullosa (EB) is associated with numerous oral manifestations, including recurrent blistering, scarring, microstomia, ankyloglossia, obliteration of the oral sulci, perioral constriction, advanced periodontitis, alveolar bone resorption, maxillary hypoplasia with mandibular prognathism, and an increased risk of oral carcinoma. Even routine oral hygiene practices, such as tooth brushing, may precipitate bullae formation on the oral mucosa [10,11]. 

Systemic features include recurrent blisters, erosions, and scars affecting the hands, feet, elbows, and knees, frequently resulting in joint contractures, finger deformities, and nail loss [12]. Painful oral blisters further restrict mouth opening, while finger deformities impair manual dexterity and compromise effective oral hygiene.

Prosthetic rehabilitation of patients with microstomia presents challenges at every stage, ranging from preliminary impression making to prosthesis fabrication.

Adequate mouth opening is essential during impression procedures to ensure accurate tray placement and alignment. In patients with EB, restricted oral opening combined with mucocutaneous fragility requires modification of standard impression techniques. This manuscript describes an innovative impression approach for RPD rehabilitation in a partially edentulous patient with microstomia, skin and mucosal fragility, and finger deformities associated with EB.

## Case presentation

**Figure 1:**
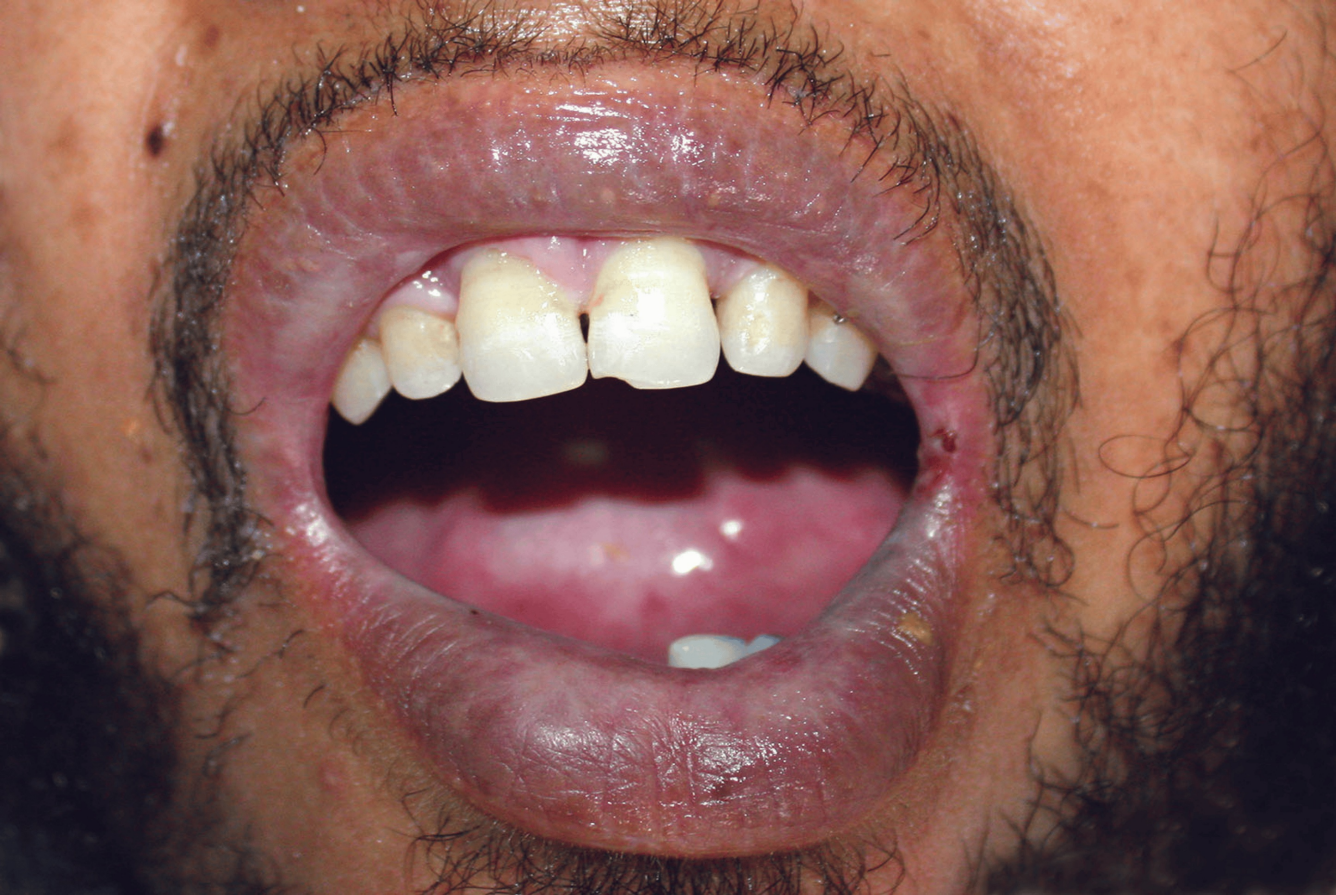
Extraoral view showing microstomia

A 37-year-old partially edentulous man with microstomia (Figure 1) secondary to EB presented for prosthetic rehabilitation at the prosthodontic unit of the Dental Center of Treatment and Diagnosis, Ibn Sina Hospital, Rabat.

The patient had been diagnosed with EB more than eight years earlier and was under regular medical management. General physical examination revealed microstomia, facial skin induration, thinning of lips with reduced mobility, and thin, dry oral mucosa. The interincisal distance was approximately 20 mm, based on clinical estimation at the time of examination.

Microstomia, defined as a chronic reduction in oral aperture diameter, was associated with progressive skin dryness. Its gradual development was attributed to repeated wound-healing processes leading to keloid formation and fibrous scar bands.

**Figure 2:**
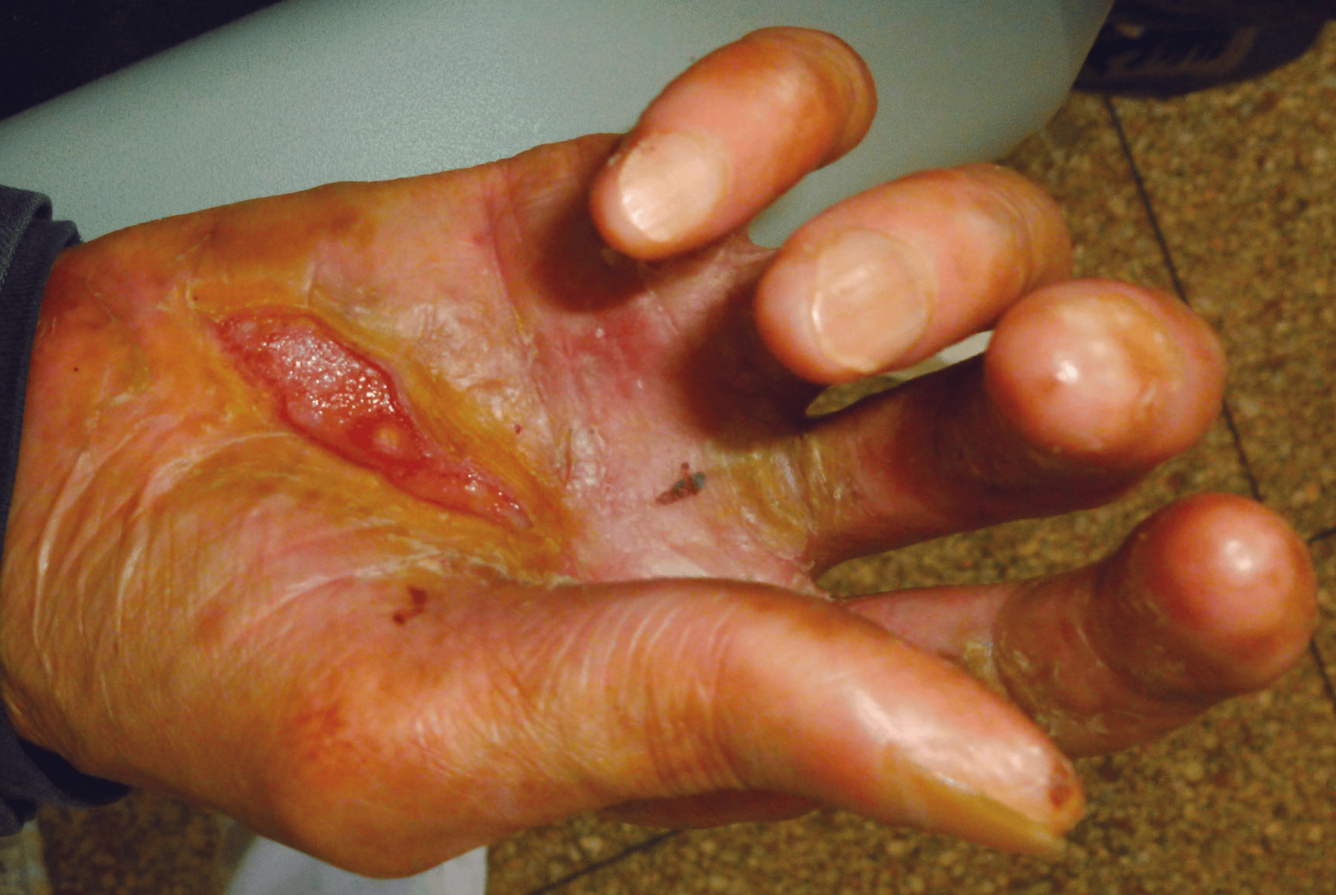
Wounds and scarring on the palmar surfaces of the hands

The patient’s extremities were also affected, with wounds and scarring particularly apparent on the palmar surfaces of the hands (Figure 2). Digital adhesions were present, and partial nail loss was observed.

Moreover, the patient had experienced tooth loss secondary to dental caries, as the restricted oral opening rendered restorative dental procedures challenging.

Technique 

Because of the restricted mouth opening, prosthetic treatment was challenging, especially during impression procedures. Consequently, all clinical steps were performed after generous lubrication of the lips with petroleum jelly. The lubricant was applied circumorally to moisten the oral mucosa, thereby facilitating lip manipulation, and was reapplied as needed throughout the procedure. 

Preliminary impression

**Figure 3:**
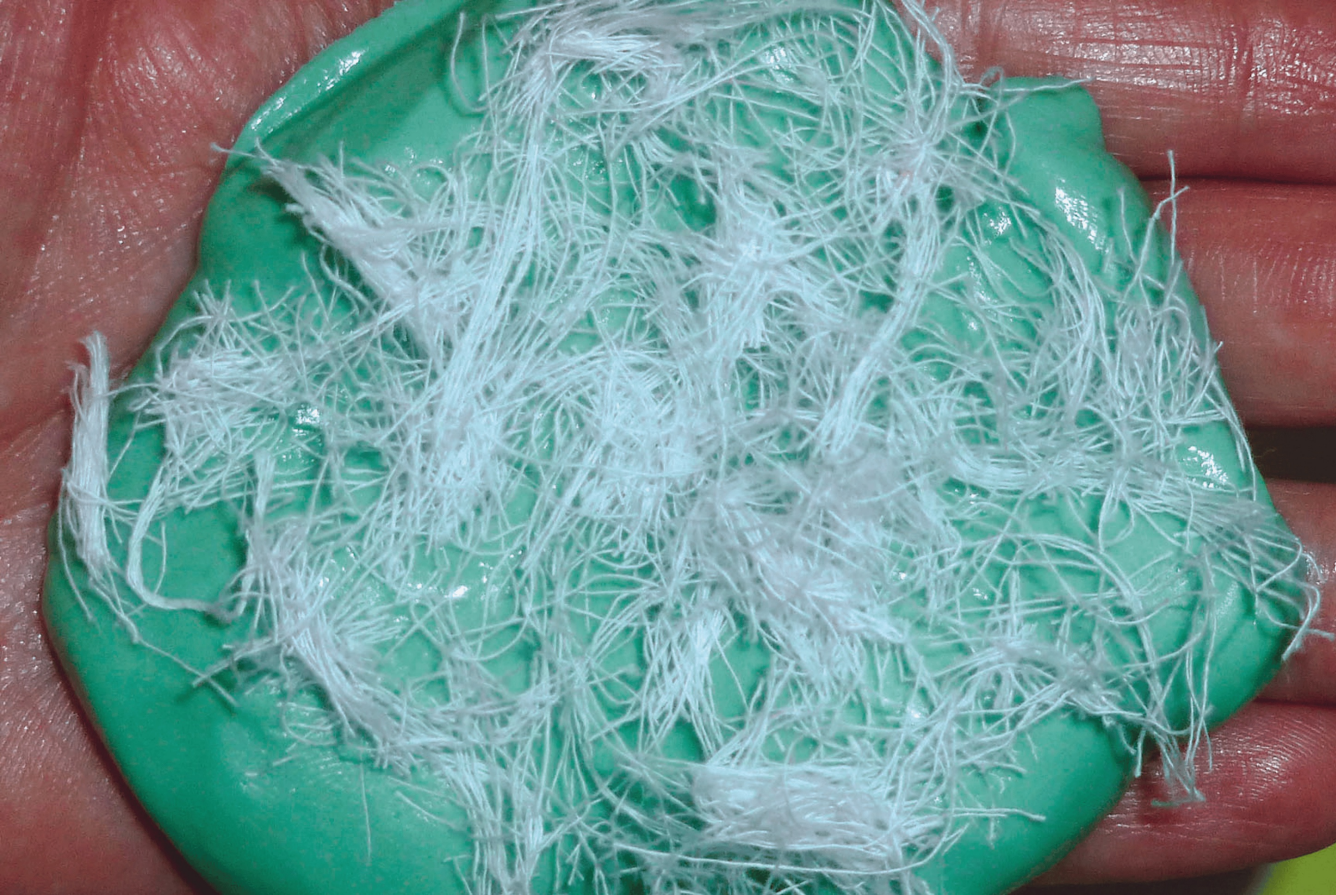
Incorporation of compressive fibers into the impression material to reinforce the preliminary impression

Conventional stock trays could not be used due to the restricted oral opening. Therefore, the preliminary impression was obtained using heavy-body silicone putty reinforced with compressive fibers (Figure 3).

**Figure 4:**
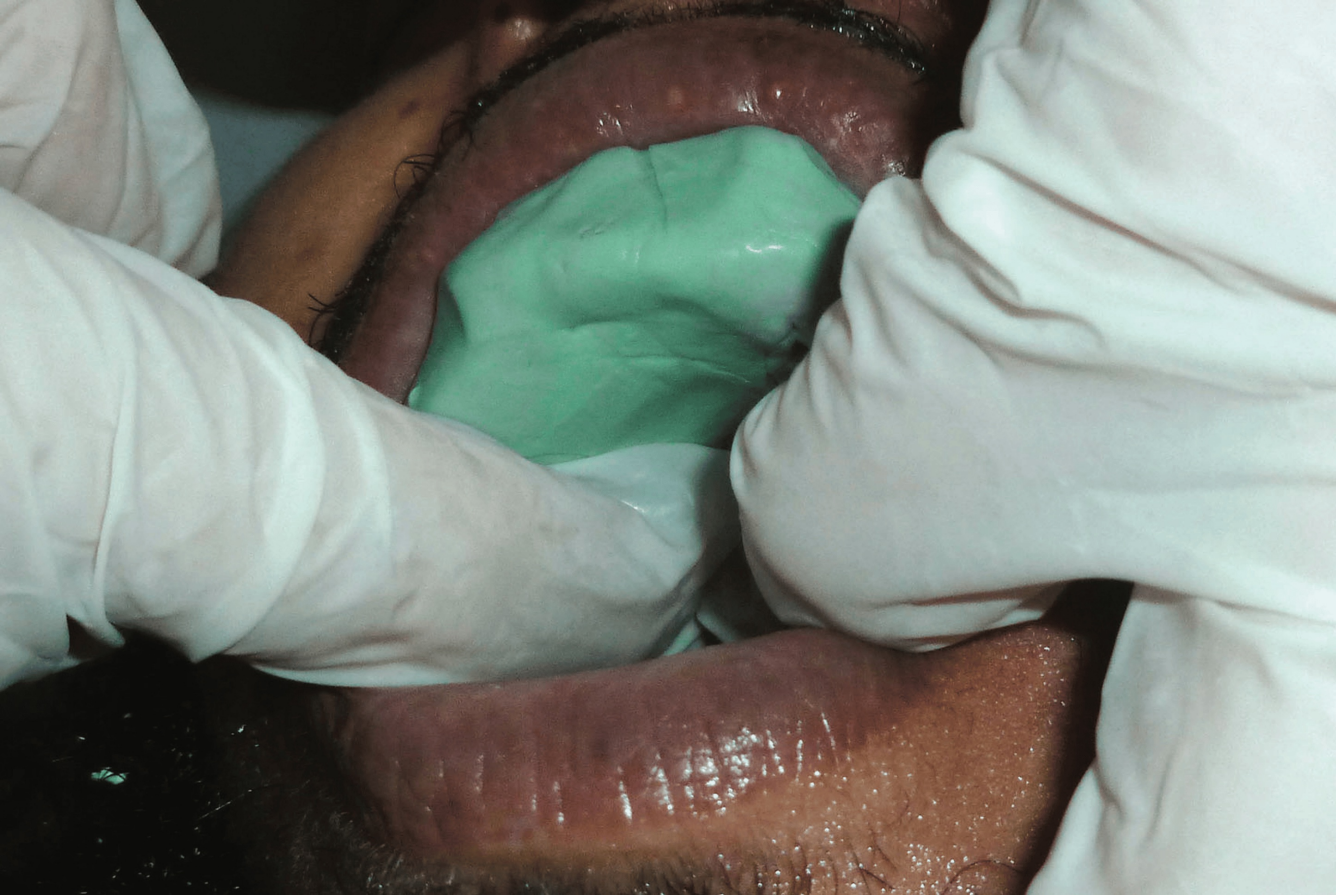
Preliminary impression obtained using heavy-body silicone putty, digitally molded and applied intraorally

Once positioned intraorally, the impression material was meticulously molded over all essential anatomical regions using a combination of functional and manual manipulation (Figure 4).

**Figure 5:**
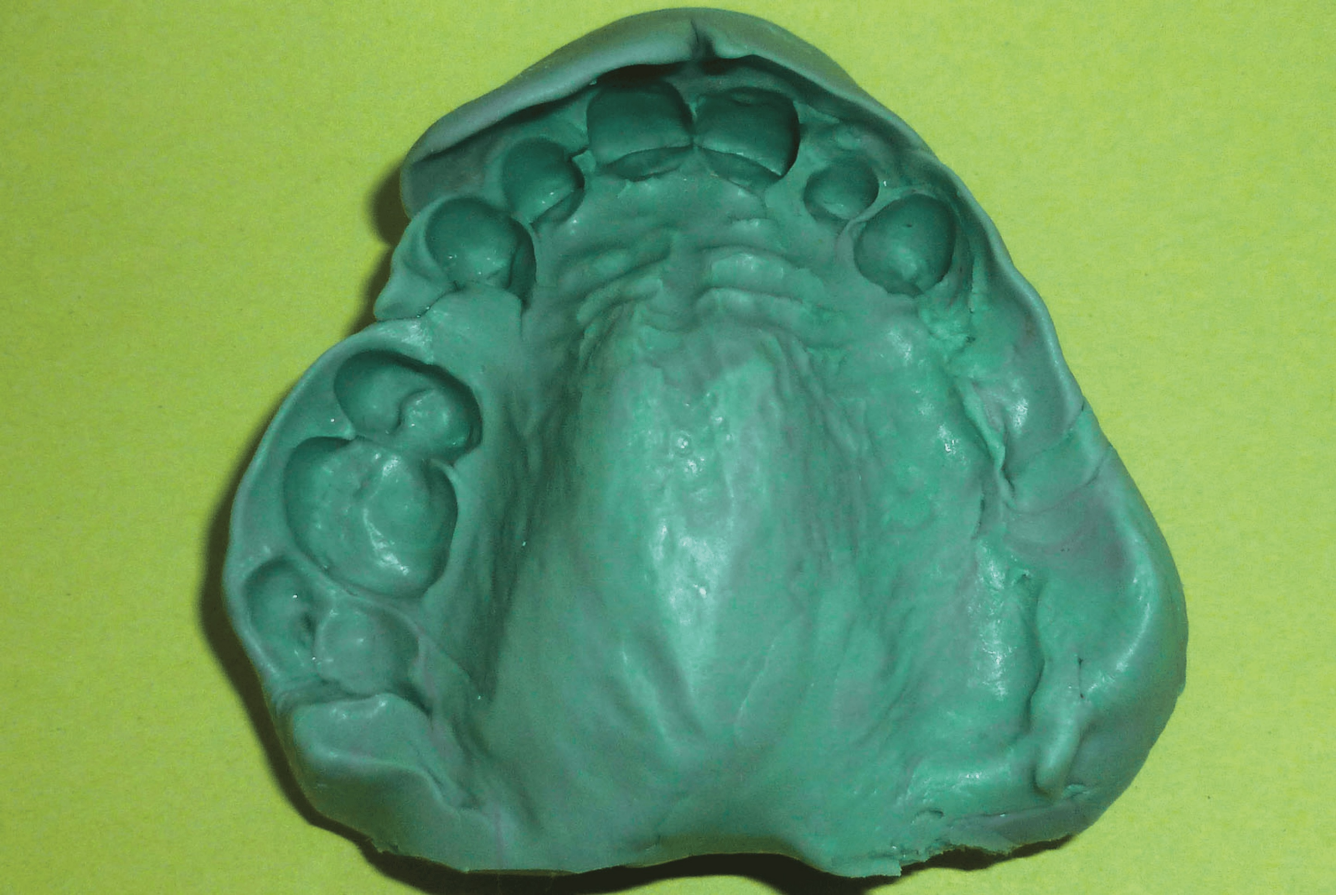
A preliminary maxillary impression was obtained using heavy-body silicone putty, allowing atraumatic removal

The flexibility of the material allowed atraumatic removal of the impression (Figure 5).

*Fabrication of Custom Tray* 

Prior to the secondary impression, all necessary preprosthetic procedures were completed, and the custom tray was tried intraorally.

**Figure 6:**
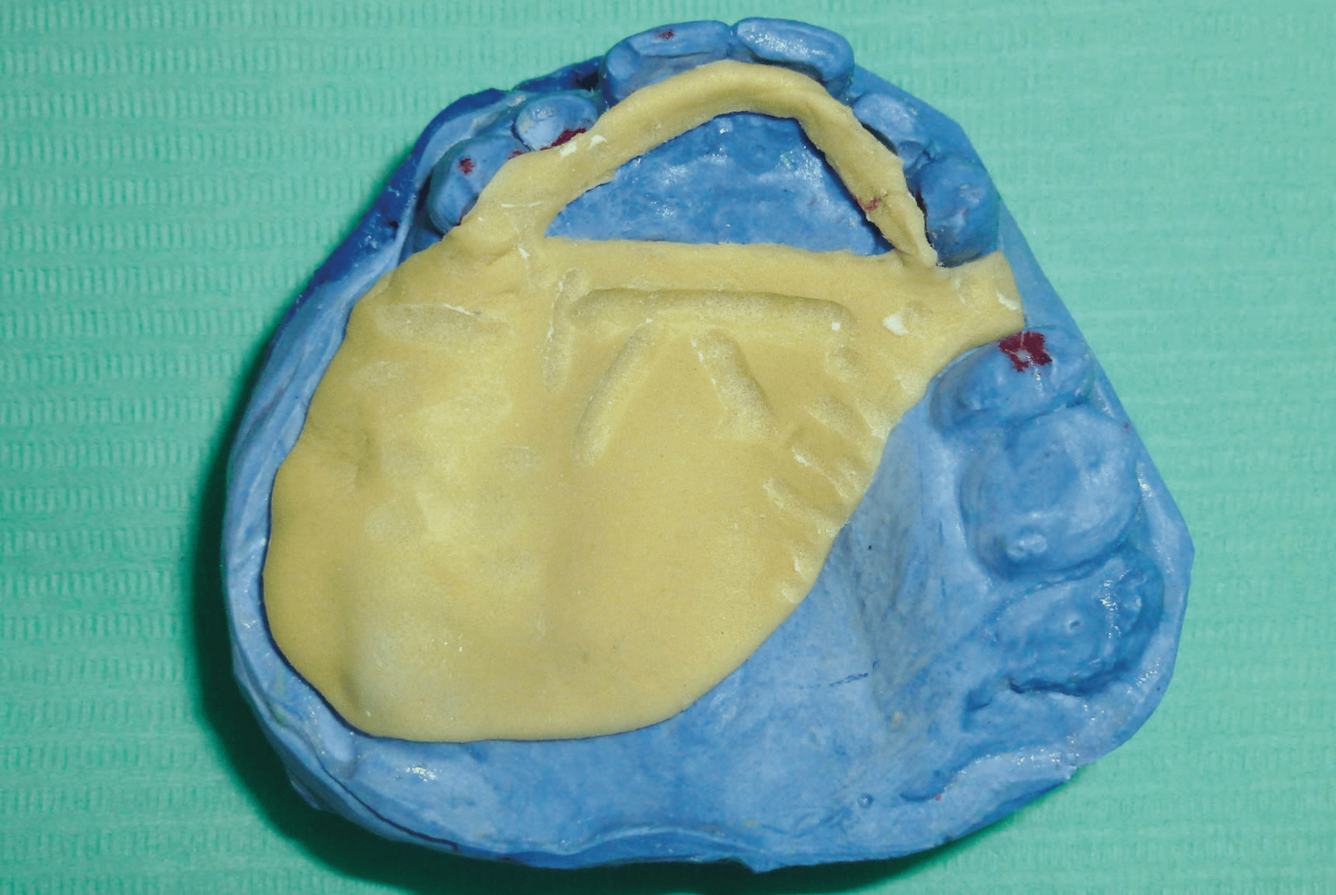
Maxillary custom tray (part 1): rigid support fabricated from self-curing acrylic resin

**Figure 7:**
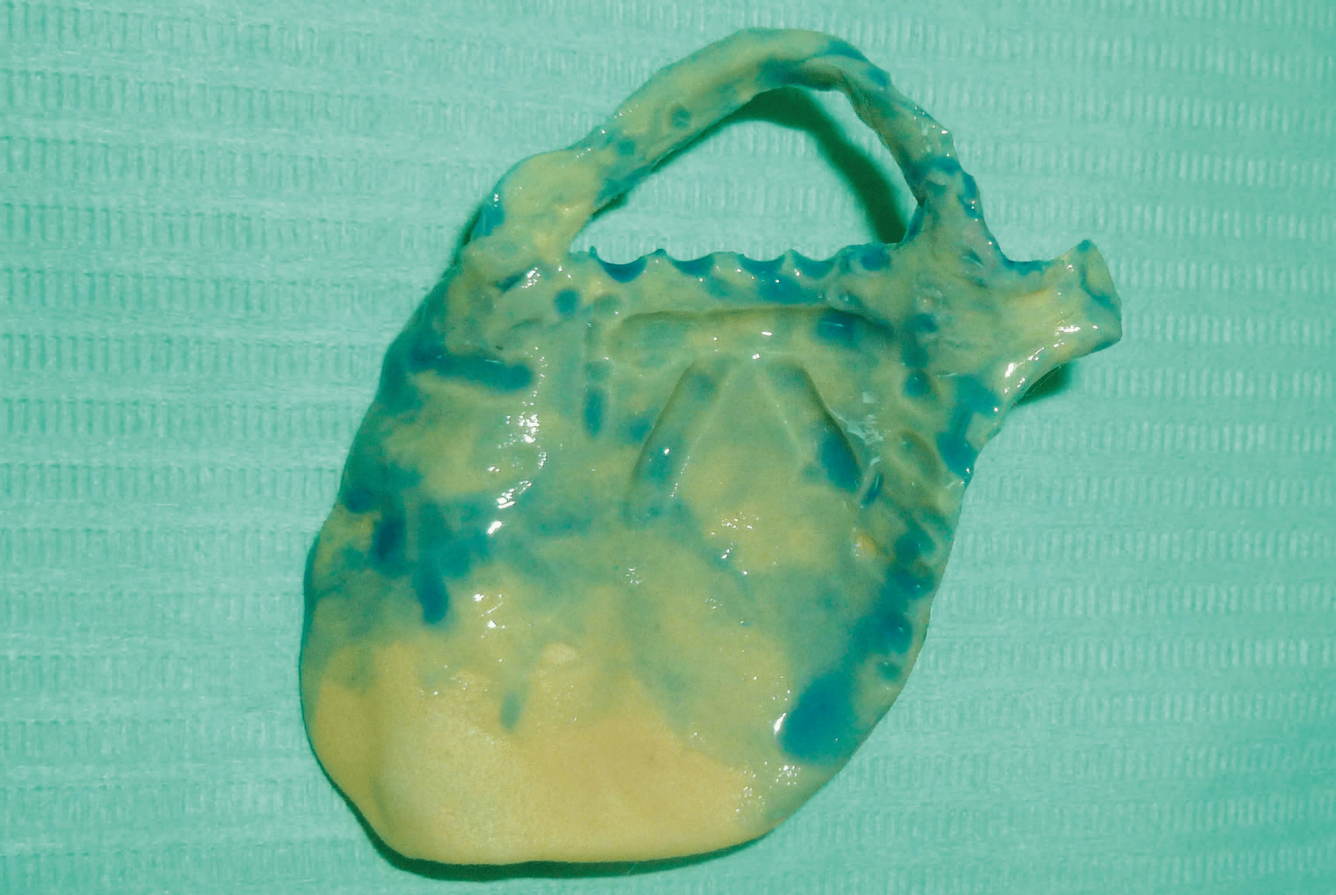
Application of adhesive on the upper surface of the resin framework

The custom tray consisted of two components:

Part 1: the first component was fabricated on the primary cast and served as a rigid support made of self-curing resin (FORMATRAY®, Zhermack SpA, Badia Polesine, Italy), reproducing the configuration of the planned prosthesis. This resin framework rested on the edentulous ridges and palatal surfaces of the anterior teeth. It was designed without a handle and did not cover teeth that would not support the prosthesis. Retention grooves were created on its upper surface and coated with adhesive (Figures 6, 7).

**Figure 8:**
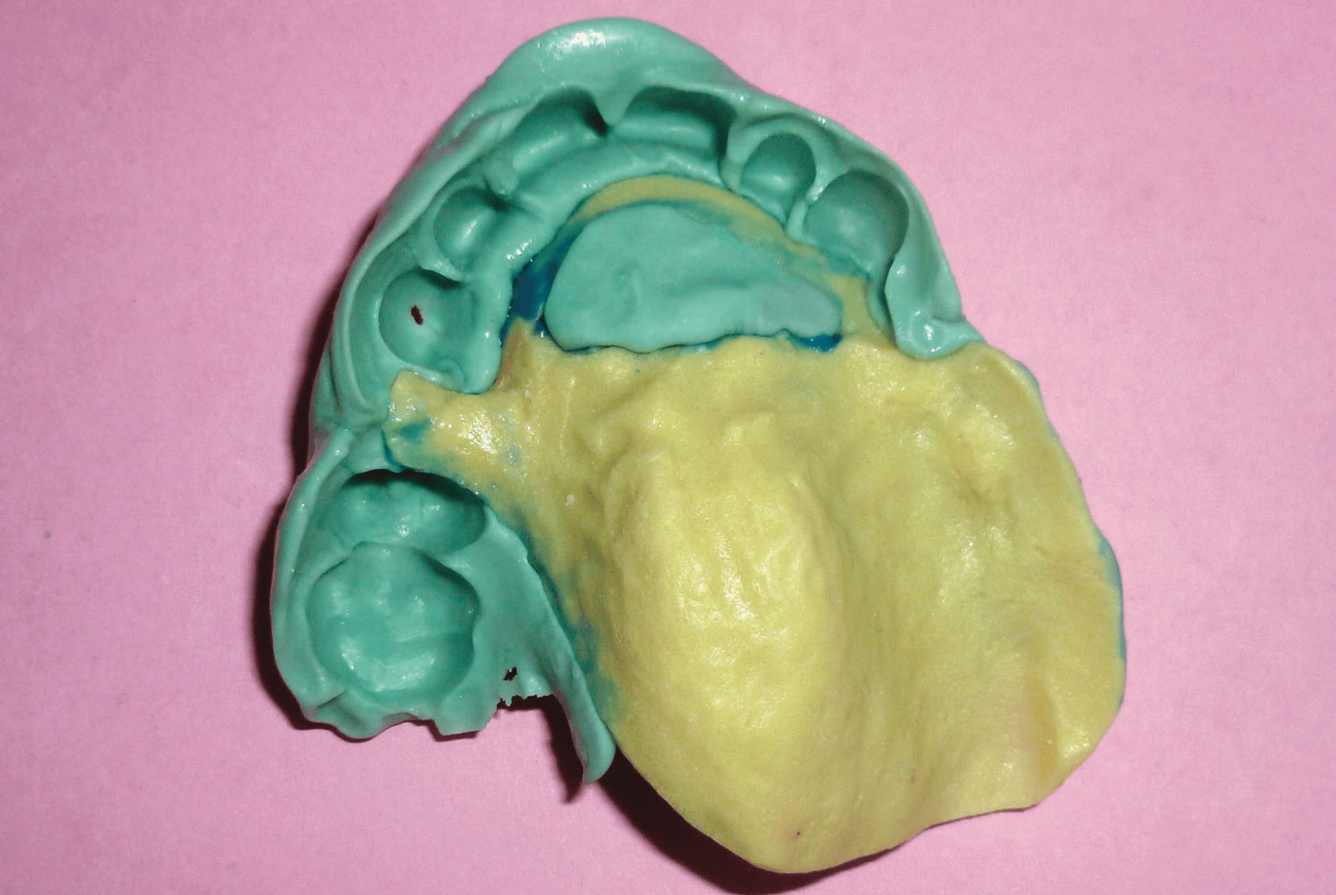
Maxillary custom tray (part 2): heavy-body silicone component designed to capture the abutment teeth

Part 2: the second component consisted of heavy silicone forming a semi-flexible overlay. This portion encompassed the abutment teeth of the future prosthesis and engaged the retention grooves of the resin framework (Figure 8).

Secondary Impression

**Figure 9:**
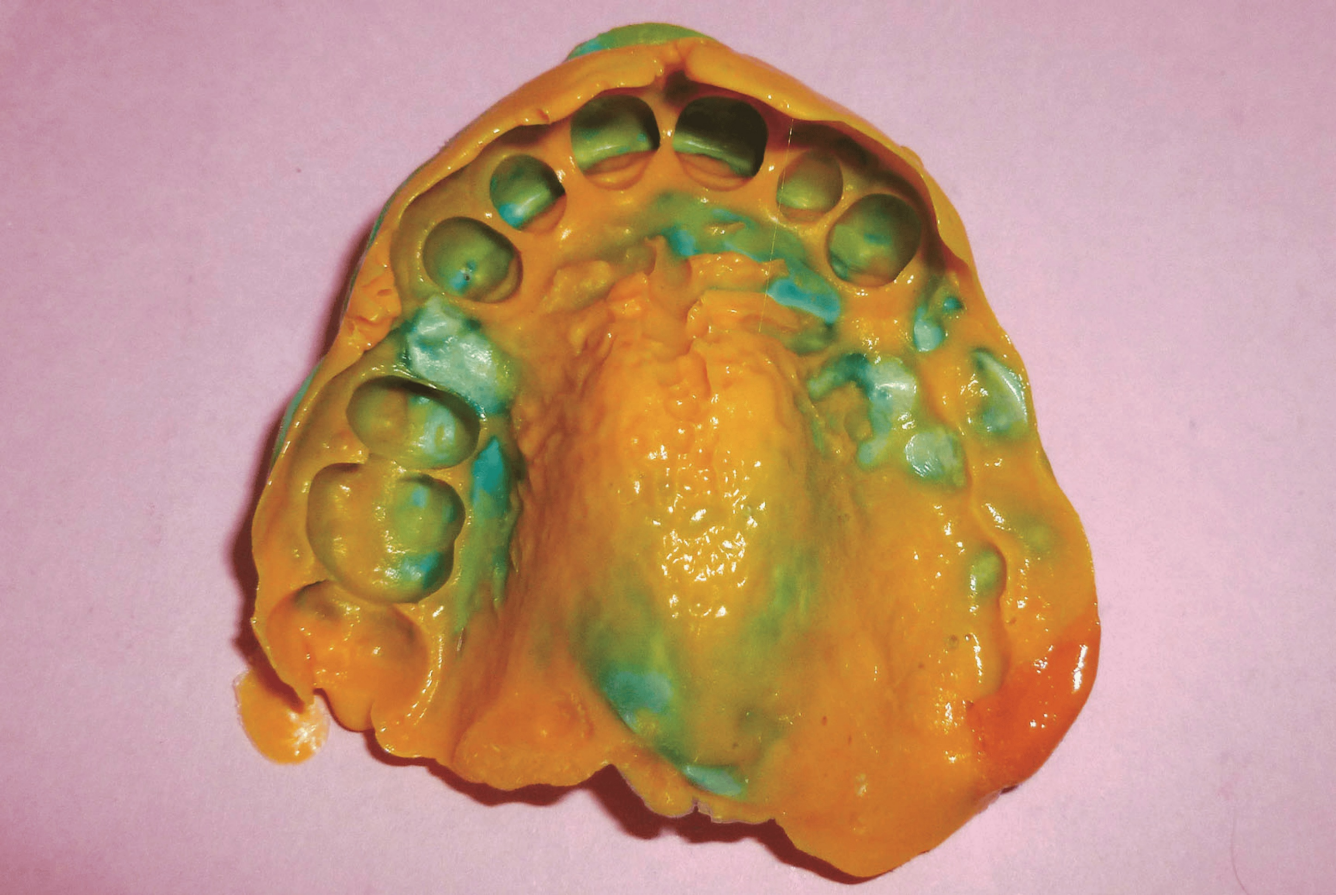
Recording of the entire arch using low-viscosity C-silicone material (ORANWASH, Zhermack SpA)

After preparation of the silicone tray and removal of the interdental fins using a scalpel blade, the functional impression was obtained in a single step with the individual impression tray using a low-viscosity C-silicone material (ORANWASH®, Zhermack SpA) (Figure 9).

**Figure 10:**
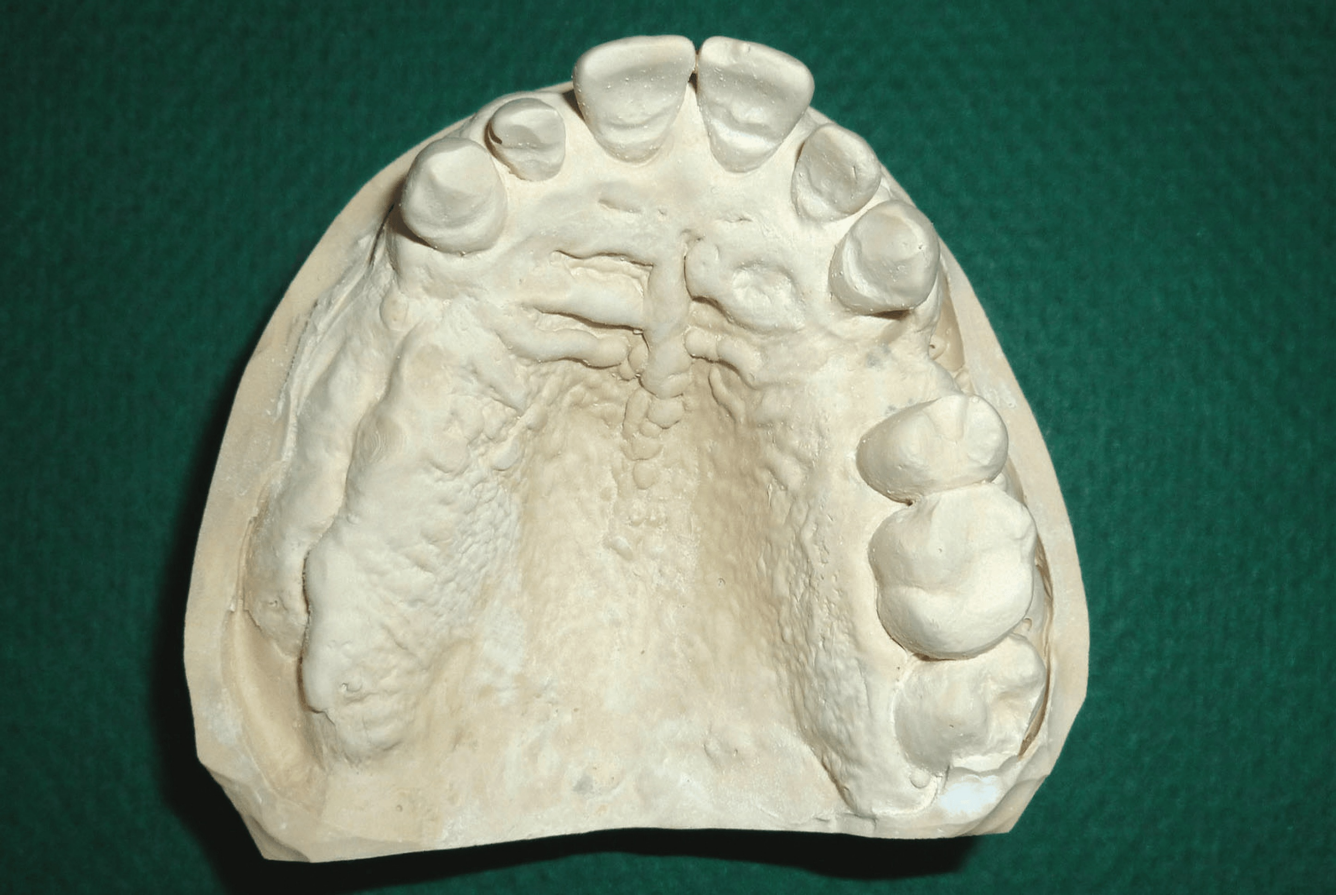
Definitive maxillary master cast showing the denture-bearing areas recorded during the final impression

The impression was subsequently poured in dental stone to obtain the master cast (Figure 10). 

Following completion of the definitive impression, the RPD was fabricated using conventional prosthodontic procedures. 

The patient was followed for two years after prosthetic delivery, during which the prosthesis remained functional and comfortable, with no major complications and favorable long-term tolerance.

## Discussion

Several prosthodontic treatment approaches have been described for patients with microstomia. These prostheses include sectional, magnetic, and collapsible prostheses. Various connecting mechanisms, such as hinges, pins, attachments, and clasp retainers, have also been reported [13].

However, such designs may exhibit limited structural durability and do not always provide adequate retention or stability during mastication. Furthermore, manipulation of these prostheses can be challenging, as many affected patients present with hand deformities [14].

In the present case, fabrication of a single-piece RPD was selected as the treatment approach, as the patient declined more complex prosthetic solutions.

Prosthetic rehabilitation in those with microstomia is challenging at every stage, from preliminary impression making to prosthesis fabrication. Numerous impression tray designs have been described in the literature. In this case, a flexible tray reinforced with compressive fibers was successfully used for the preliminary impression. 

To achieve a more precise and functional impression, a custom tray was fabricated and adapted to both the patient’s clinical condition and the specific characteristics of edentulism. 

This technique offers several advantages, indicating a reasonably feasible fabrication process, accurate intraoral positioning with adequate stability, optimal impression material thickness, and the absence of complex or expensive auxiliary systems. It reduces patient trauma and intraoral bulk, relies on material readily available in the dental office, even in emergencies, and minimizes the risk of distortion owing to the rigidity of the resin framework.

However, this technique remains time-consuming, may be challenging to apply in the presence of bilateral posterior teeth, and requires a certain level of clinical expertise for the fabrication and handling of the impression tray.

## Conclusions

This innovative impression technique described may serve as an alternative approach for denture impression making in patients with severe microstomia. The use of a mixed custom tray composed of an acrylic framework combined with a semi-flexible overlay can be reliably employed for RPD rehabilitation in those who are partially edentulous with restricted mouth opening. However, further clinical experience and additional studies are required to validate the broader applicability of this technique.
